# Réparation chirurgicale de l´insuffisance tricuspide associée à une valvulopathie du cœur gauche: à propos de 162 cas

**DOI:** 10.11604/pamj.2021.40.259.24146

**Published:** 2021-12-23

**Authors:** Amine Majdoub, Salaheddine Boulmakoul, Anas Elhafidi, Mohammed Messouak

**Affiliations:** 1 Service de Chirurgie Cardio-Vasculaire, CHU Hassan II Fès, Université Sidi Mohamed Ben Abdallah, Faculté de Médecine, de Pharmacie et de Médecine Dentaire, Fès, Morocco

**Keywords:** Valve tricuspide, anneau, De Vega, dysfonction ventricule droit, Tricuspid valve, ring, De Vega, right ventricular dysfunction

## Abstract

L´insuffisance tricuspide (IT) constitue un facteur pronostic dans l´évolution des patients opérés d´une valvulopathie du cœur gauche. L´objectif de cette étude est d´évaluer les résultats postopératoires de l´insuffisance tricuspide associée à une valvulopathie du cœur gauche et de déterminer les facteurs liés à l´échec de la réparation chirurgicale. Une étude rétrospective incluant 162 patients a été menée durant une période allant du mois de janvier 2009 au mois de juillet 2019 incluant tous les patients opérés pour une IT associée à une chirurgie valvulaire gauche. L´âge moyen de nos patients était de 39,70 ans ± 10,8 avec une prédominance féminine. Sur les 162 plasties tricuspidiennes, nous avons effectué 47 (29%) annuloplasties prothétiques, 103 (63,5%) annuloplasties de DeVega, et 12 (7,5%) réductions ou effacement de l´anneau postérieur. Sur le plan échocardiographique, l´évolution a été marquée par une nette amélioration des moyennes des différents paramètres morphologiques et fonctionnels, pourtant l´échec de la plastie tricuspide était présent chez 24 (14,8%) patients de tous les opérés. Nous avons colligé 4 décès dans les 30 jours postopératoires soit un taux de mortalité hospitalière de 2,46%. Les causes du décès sont réparties en dysfonction ventriculaire droite réfractaire pour 2 cas, et en dysfonction ventriculaire gauche sévère pour 2 cas. Les facteurs liés à l´échec de la plastie tricuspide dans notre travail sont: l´insuffisance tricuspide préopératoire sévère, des pressions pulmonaires postopératoires supérieures à 60mmHg, le rapport VD/VG télé diastolique préopératoire supérieur à 0,6. Les résultats de la présente étude permettent de mieux comprendre l´évolution des patients subissant une chirurgie de l´insuffisance tricuspide et apportent des informations permettant d´évaluer de façon plus fiable les indications des plasties tricuspides.

## Abstract (en)

Tricuspid regurgitation (TR) is a prognostic factor associated with the outcome of patients undergoing surgical treatment of left valvular heart disease. The purpose of this study was to evaluate the postoperative outcome of patients with tricuspid regurgitation associated with left valvular heart disease and to identify factors associated with surgical failure. We conducted a retrospective study of 162 patients over the period January 2009 to July 2019. The study included all patients undergoing surgical treatment of TR and left valve repair. The average age of patients was 39.70 years ± 10.8, with a female predominance. We performed 47 (29%) tricuspid prosthetic annuloplasties, 103 (63.5%) De Vega´s annuloplasties and 12 (7.5%) posterior ring reductions or replacements out of 162 tricuspid valve repairs. Patients´ outcome was marked by clear improvement in morphological and functional echocardiographic parameter means. Surgical failure was reported in 24 (14.8%) patients. Four patients died within 30 postoperative days, reflecting a hospital mortality rate of 2.46%. The causes of death were refractory right ventricular failure (2 cases), and severe left ventricular failure (2 cases). Factors related to failure of tricuspid repair were: severe pre-operative tricuspid failure, postoperative pulmonary hypertension (> 60mmHg) and preoperative RV/LV ratio > 0.6. The results of this study provide a better understanding of the outcomes of patients undergoing surgical treatment of tricuspid failure and provide information on the indications for surgery.

## Introduction

L´insuffisance tricuspide constitue une pathologie fréquente. Elle est le plus souvent fonctionnelle. La régurgitation résulte de la dilatation des cavités droites et de l´anneau tricuspidien secondaire à une surcharge mécanique du ventricule droit, le plus souvent par hypertension pulmonaire, quelle qu´en soit l´origine: les lésions mitrales ou mitro-aortiques sont fréquemment en cause [1], mais aussi plus rarement les insuffisances ventriculaires gauches hypertensives ou ischémiques, les cardiomyopathies, les cœurs pulmonaires aigus ou chroniques, emboliques ou par insuffisance respiratoire, et l´hypertension artérielle pulmonaire primitive [2,3].

Plusieurs études ont démontré que le pronostic des patients opérés dépend de l´évolution de la fuite tricuspide [1], ainsi, une insuffisance tricuspide résiduelle pourrait être responsable de la persistance des signes droits et de la nécessité d´une prescription des diurétiques ou même dans quelques cas une réintervention chirurgicale sur la valve tricuspide.

Le but de cette étude est d´évaluer les résultats postopératoires de la chirurgie de l´insuffisance tricuspide associée à une atteinte valvulaire du cœur gauche et de déterminer les facteurs liés à l´échec de la réparation tricuspide.

## Méthodes

**Conception et cadre de l´étude:** il s´agissait d´une étude monocentrique, rétrospective et analytique réalisée au service de chirurgie cardiovasculaire du Centre hospitalier universitaire Hassan II de Fès (Maroc).

**Population étudiée:** nous avons inclus tous les patients qui ont bénéficié d´un geste chirurgical sur la valve tricuspide associé à une chirurgie valvulaire gauche durant une période allant du mois de septembre 2009 au mois de juillet 2019. Au total, 162 patients opérés ont été étudiés. Avec exclusion des malades qui ont bénéficié d´une plastie de la valve tricuspide associée à une correction d´une pathologie congénitale (communication interauriculaire, communication interventriculaire, canal atrio-ventriculaire, tétralogie de Fallot..). Les opérés dont les données colligées se sont avérées insuffisantes à l´exploitation sont exclus également.

**Collecte des données:** nous avons relevé les caractéristiques cliniques et échographiques préopératoires ainsi que les données postopératoires. Le recueil des données per opératoires a été réalisé à partir des cahiers de compte rendu opératoires et des cahiers de circulation extracorporelle. Le recueil des données pré et postopératoires a été effectué à partir des dossiers médicaux archivés. Le recueil des données échocardiographiques était basé sur les comptes rendus rédigés pas les cardiologues du centre hospitalier.

**Définitions:** la réparation tricuspide a consisté soit en une plastie de De-Vega soit à une annuloplastie par un anneau de Carpentier-Edwards. Une insuffisance tricuspide post opératoire significative (échec de la plastie) a été définie par une fuite tricuspidienne grade 3 ou 4. Les variables telles que les données cliniques, les paramètres échocardiographiques pré et post opératoires, et les gestes opératoires effectués ont été prises en compte pour une analyse univariée puis multivariée.

**Analyse statistique:** les variables quantitatives sont exprimées en moyenne ± écart type. Les variables qualitatives sont exprimées en nombre (n) et pourcentage (%). La comparaison des moyennes a été effectuée en utilisant le test de student. Les variables qualitatives sont comparées soit par le test de Khi-deux de Pearson, soit par un test exact de Fischer pour les évènements rares. Une analyse multivariée par régression logistique a été réalisée afin de rechercher les facteurs liés à l´échec de la plastie tricuspide. Les variables utilisées pour l´analyse multivariée ont été incluses après l´analyse univariée quand une association entre la variable et l´échec de la plastie tricuspide a été calculée avec un p value < 0.2. Une valeur de p<0.05 a été retenue comme statistiquement significative. Toutes les données recueillies sont codées, saisies et analysées par le logiciel EPI INFO (OMS).

**Considérations éthiques:** un consentement oral a été obtenu de tous les patients avant la chirurgie. Les patients ont été informés des risques de la chirurgie et de l´intérêt de l´étude. Les dossiers des patients ont été examinés avec la plus grande confidentialité.

## Résultats

**Caractéristiques générales de la population étudiée:** l´étude a concerné 162 patients d´âge moyen de 39,70 ans ± 10,8 avec des extrêmes allant de 19 ans à 62 ans. Ils se répartissent en 112 femmes (69,1%) et 50 hommes (30,9%). Le sexe ratio (H/F) était de 0,44.

**Comorbidités et caractéristiques cliniques:** dans l´histoire clinique de nos patients, la notion de rhumatisme articulaire aigüe (RAA) a été retrouvée chez 59,2% (n=96) des patients. Plus de dix (10,5)% (n=17) des patients ont bénéficié d´un geste cardiaque dans leurs antécédents. Sept malades de notre série avaient un antécédent d´une valvuloplastie mitrale percutanée, 8 patients avaient un antécédent de commissurotomie mitrale à cœur fermé, et 2 patients avaient un antécédent de remplacement valvulaire aortique (Tableau 1). Aucun malade n´a eu auparavant un geste chirurgical sur la tricuspide. Sur le plan clinique, 87,6% (n=142) des patients avaient une dyspnée stade 3 ou 4 selon la classification NYHA, 32,7% (n=53) des malades ont présenté des signes cliniques d´insuffisance cardiaque droite.

**Tableau 1:** données cliniques et échocardiographiques préopératoires

	Pourcentages n (%) ou moyennes	Écart type	Min-Max
Age (ans)	39,7	10,8	19-62
Sexe ratio	0,44	-	-
**Antécédents**			
RAA	96(59,2%)	-	-
Antécédents d´intervention cardiaque	17(10,5%)	-	-
VMP	7(4,3%)	-	-
CCF	8(4,9%)	-	-
RVAo	2(0,7%)	-	-
**Signes cliniques**			
Dyspnée stade III ou IV	142(87,6%)	-	-
Insuffisance cardiaque droite	53(32,7%)	-	-
RHJ	49(30,2%)	-	-
TSVJ	50(30,8%)	-	-
Hépatomégalie	14(8,6%)	-	-
Ascite	3(1,8%)	-	-
**Echocardiographie préopératoire**			
AT (mm)	36,42	5,8	24-54
VD (mm)	27,1	6,49	16-47
DAP(OG) (mm)	58,75	10,01	38-92
Surface (OG) (cm^2^)	45,12	14,39	22-89
DTSVG (mm)	35,41	7,93	24-66
DTDVG (mm)	51,92	9,63	30-80
VD/VG	0,56	0,19	0,25-1,34
FEVG (%)	58,88	7,49	35-80
PAPs (mmHg)	64,02	23,52	25-140
IT (grade)	2,59	1,18	1-4

RAA=rhumatisme articulaire aigu ; VMP=valvuloplastie mitrale percutanée ; CCF=commissurotomie à cœur fermé ; RVAo=remplacement de la valve aortique ;RHJ=reflux hépato jugulaire ;TSVJ=turgescence spontanée des veines jugulaire ;AT=anneau tricuspide ; ; VD =ventricule droit ;DAP=diamètre antéro-postérieur ;OG=oreillette gauche ; DTSVG =diamètre télésystolique du ventricule gauche ;DTDVG =diamètre télédiastolique du ventricule gauche ; VG =ventricule gauche ; FEVG=fraction d´éjection du ventricule gauche ; PAPs =pression artérielle pulmonaire systolique ; IT =insuffisance tricuspidienne.

**Figure 1:**
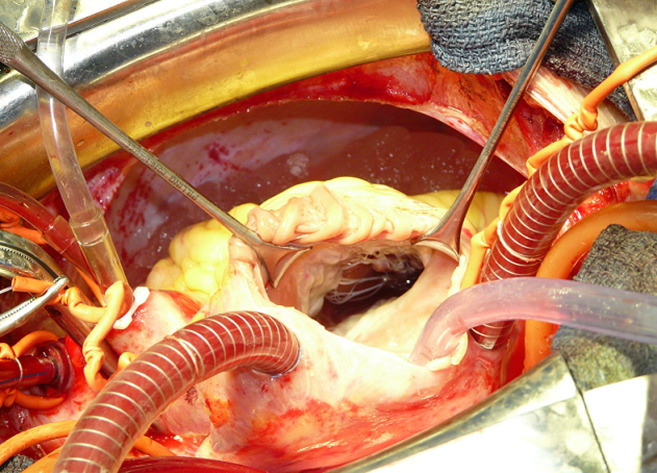
vue opératoire valve tricuspide avec anneau dilaté

**Figure 2:**
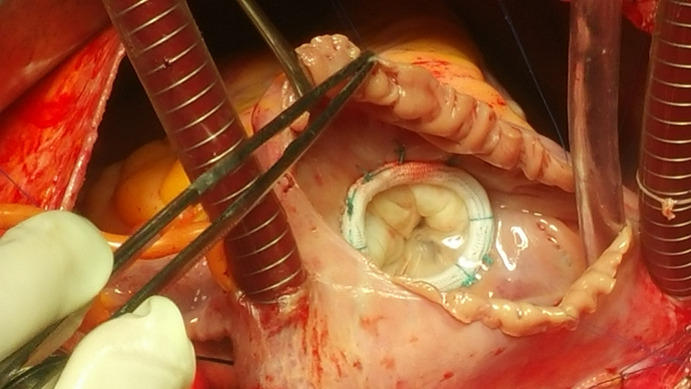
vue opératoire après mise en place d´un anneau tricuspide 3D

**Données paracliniques et opératoires:** les données échocardiographiques préopératoires sont résumées dans le Tableau 1. Plus de la moitié des malades (n=88) avaient une IT grade 3 ou 4 avec des PAPS allant de 25 à 140 mmHg, 51,8% (n=84) des patients avaient des PAPS > 60 mmHg, 13,5% (n=22) dépassaient 80 mmHg et 9,2% (n=15) avaient des PAPS > 100 mmHg. Le diamètre de l´anneau tricuspide moyen était de 36,42 mm avec des extrêmes de 24mm à 54mm, 79% (n=128) des malades avaient un diamètre tricuspide supérieur à 32mm Figure 1. Le quart de nos patients (n=44) avait un ventricule droit dilaté (VD/VG> 0,6) avec un rapport VD/VG télé diastolique moyen de 0,56. La fraction d´éjection du ventricule gauche (FE VG) moyenne était de 58,88% ±7,49, 12,3% (n=20) des patients avaient une FE < 50%. Les gestes opératoires réalisés ont été conservateurs chez tous les patients. De ce fait, aucun remplacement valvulaire tricuspide n´a été effectué et la totalité des gestes effectués ont été associés à un geste chirurgical mitral ou mitro-aortique. Sur les 162 plasties tricuspides, nous avons effectué 47 (29%) annuloplasties prothétiques par anneau tridimensionnels Figure 2, 103 annuloplasties de DeVega (63,5%), et 12 réductions de l´anneau postérieur (7,5%). La durée moyenne de la CEC était de 93 min ± 22 avec des extrêmes allant de 45 à 188 min. Le sevrage de la CEC était difficile pour 13,5% (n=22) de nos patients. Le contrôle échocardiographique hospitalier (< 30 jour postopératoire) est résumé dans le Tableau 2.

**Tableau 2:** données échocardiographiques pré et postopératoires

	Données échographiques préopératoires	Données échographiques postopératoires	p Value
**Anaslyse morphologique**			
VD (mm)	27,1	24,97	0,24
DAP(OG)(mm)	58,75	50,74	0.18
DTSVG (mm)	35,41	34,6	0,29
DTDVG (mm)	51,92	49,91	0,31
Analyse fonctionnelle			
PAPs (mmhg)	64,02	39,1	0.007
Grade IT	2,59	1,28	0.01
Fonction ventriculaire gauche			
FE %	58,88	55,8	0,19

VD =ventricule droit ; DAP=diamètre antéro-postérieur ; OG=oreillette gauche ; DTSVG =diamètre télésystolique du ventricule gauche ; DTDVG =diamètre télédiastolique du ventricule gauche ; PAPs =pression artérielle pulmonaire systolique ; IT =insuffisance tricuspidienne ; FE=fraction d´éjection

**Evaluation des données post opératoires:** l´évolution a été marquée par une amélioration significative de la PAPs et le grade de l´IT, ainsi que le pourcentage des malades qui avaient une HTAP importante (sup à 60mmgh) est passé significativement de 51,80% (n=84) en préopératoire à 9,2% (n=15) en postopératoire (p value<0.001), même évolution a été marquée pour le pourcentage des patients qui avaient un rapport télé diastolique VG/VD supérieure à 0,6 qui est passé de 27% (n=44) à 16% (n=26) (p value=0,01). L´évolution de la fuite tricuspide est résumée dans le Tableau 3.

**Tableau 3:** évolution de la fuite tricuspide en postopératoire

Statut préopératoire		Statut postopératoire	
	N		N
**IT Grade 1 + Grade 2**	74	IT Minime ou absente	**70**
		IT Grade 2	**3**
		IT Grade 3	**1 DCD**
		IT Grade 4	**0**
**IT Grade 3**	49	IT Minime ou absente	**35**
		IT Grade 2	**8**
		IT Grade 3	**6**
		IT Grade 4	**0**
**IT Grade 4**	39	IT Minime ou absente	**10**
		IT Grade 2	**9**
		IT Grade 3	**13**
		IT Grade 4	**4**
		DCD	**3**

IT =insuffisance tricuspidienne; DCD : décédé

Nous avons considéré comme échec toute IT postopératoire moyenne ou sévère, dans le groupe des malades qui avaient une IT grade 1 ou 2 (avec anneau tricuspide dilaté) 1 patient (1,3%) a présenté une IT résiduelle significative en postopératoire, et parmi les 49 malades qui avaient une IT grade 3 en préopératoire, 6 cas (12%) sont sortis d´une IT résiduelle significative, alors que l´échec de la plastie tricuspide était présente chez 43,5% (17/39) des malades qui avaient une IT grade 4. Au total, on avait 24 cas (14,8%) d´échec de la plastie tricuspide pour tous les opérés. Nous avons colligé 4 décès dans les 30 jours postopératoires soit un taux de mortalité hospitalière de 2,46%. Les causes du décès sont réparties en dysfonction ventriculaire droite réfractaire pour 2 cas, et en dysfonction ventriculaire gauche sévère pour les autres 2 cas (VG à 80mm et FE à 30%).

A partir du Tableau 4, les facteurs prédictifs d´échec de la plastie tricuspide en analyse multivariée dans notre série sont: Rapport VD/VG télé diastolique préopératoire supérieur à 0,6(p=0,0071), PAPs préopératoire > à 60mmhg (p= 0,0445), IT préopératoire grade 4(p=0,0096).

**Tableau 4:** facteurs liés à l´échec de la plastie tricuspide (analyse uni et multivariée)

		Analyse univariée		Analyse multivariée	
		Nombre de patients (%)	p value	OR (IC à 95%)	p value
Age	<40ans	13/72(18 %)	0,39		
	>40ans	12/90(13 %)			
Sexe	Homme	18/112(16%)	0,23		
	Femme	6/50(12%)			
Présence de signes droits	OUI	9/53(17%)	0,29		
	NON	15/109(13,7%)			
Omi	OUI	3/17(17,6%)	0,40		
	NON	21/145(14,4%)			
Hmg	OUI	4/14(28,5%)	0,18	1,31(0.84-2,51)	0,31
	NON	20/148(13,5%)			
Anneau tricuspide	<32mm	2/34(5,8%)	0,07	1,53(1,01-3,54)	0.23
	>32mm	22/128(17,1%)			
Vd/dtdvg préopératoire	<0,6	6/117(5,1%)	0,003	7,33(5,860-16,927)	0,0071
	>0,6	18/45(40%)			
Vd/dtdvg postopératoire	<0,6	16/136(11,7%)	0,004	2,5(0,99-4,42)	0,09
	>0,6	8/26(30,7%)			
Paps préopératoire	<60 mmHg	2/78(2,5%)	0,0006	9,02(7,182-19,225)	0,0445
	>60 mmHg	22/84(26,1%)			
Paps postopératoire	<60mmhg	15/147(10,2%)	0,0005		
	>60 mmHg	9/15(60%)			
It préopératoire grade 4	OUI	18/39(46,1%)	0,002	6,87(5,727-16,493)	0,0096
	NON	6/123(4,8%)			
Geste chirurgical sur la tricuspide	DE VEGA	15/103(14,5%)	0,69		
	AT	9/47(19,1%)			
Geste opératoire associé	RVM	10/100(10%)	0,03	1,72(0.91-2,81)	0.11
	DRV	14/50(28%)			

OMI =Œdèmes des membres inférieurs ; AT =annuloplastie tricuspide (prothétique) ; VD =ventricule droit ; DTDVG =diamètre télédiastolique du ventricule gauche ; PAPs =pression artérielle pulmonaire systolique ; IT =insuffisance tricuspidienne ; RVM=remplacement de la valve tricuspide ; DRV=double remplacement valvulaire.

## Discussion

L´atteinte valvulaire tricuspide, concomitante à des lésions valvulaires mitrales et/ou aortiques, est considérée comme pourvoyeuse d´une importante morbimortalité à court et à long termes. Sa cure chirurgicale représente un grand défi pour le chirurgien et fait appel à plusieurs techniques dont le choix reste jusqu´à nos jours incertains. Le but de notre travail était d´évaluer les résultats postopératoires de l´insuffisance tricuspide associée à une valvulopathie du cœur gauche et de déterminer les facteurs liés à l´échec de la réparation chirurgicale, ceci à travers une série de cas opérés et colligés dans notre service.

L´âge moyen de nos patients était de 39,7 ans ± 10,8, il est similaire à celui d´une étude chinoise [2,3] publiée en 2007 et à celui rapporté dans des séries tunisiennes [4-6]. Notre population d´étude est moins âgée comparativement aux études Européennes et Américaines [7-9], il s´explique par la prédominance, dans notre étude, de l´étiologie rhumatismale comparée aux atteintes valvulaires dégénératives qui surviennent à un âge plus avancé. Une prédominance féminine a été retrouvée dans notre série (69,10%) et rapportée par la majorité des études publiées variant de 62 à 83% [6,9-13]. La fuite tricuspide était moyenne à sévère dans 54,3% de nos patients, dans l´étude de Bernal *et al*. [9] et de Tager *et al*. [14], ce stade de fuite était présent chez ; successivement ; 98% et 25% des malades. Conformément à la littérature, une nette prédominance de l´étiologie fonctionnelle est retrouvée [8,15,16] ; ainsi, nous avons noté, sur les données échographiques, que 79% avaient un AT > 32mm. Le calcul de la PAPs de notre série trouve une moyenne importante à 64,02mmHg, cette valeur était similaire dans d´autres séries [17,18], et moins élevée dans d´autres études [6]. La moyenne de la fraction d´éjection (FE) du ventricule gauche de nos patients était de 58,88%. Cette valeur est très proche de celles retrouvées dans les autres études [6,11,19]. 50 patients (30,8%) de notre série ont eu une triple chirurgie valvulaire et 112 patients (69,1%) ont eu une chirurgie mitro tricuspide. Les gestes chirurgicaux réalisés sur la valve tricuspide de notre série étaient tous des gestes conservateurs à 100%. L´annuloplastie tricuspide de DeVega représente le geste conservateur le plus fréquemment réalisé dans notre série (63,58%), suivi par l´annuloplastie prothétique (29,01%). Dans la littérature, l´annuloplastie de DeVega est la technique conservatrice la plus utilisée variant de 52% à 100% [4,6,11,14,20]. Dans d´autres séries c´est l´annuloplastie de Carpentier qui prédomine [5].

La durée moyenne de la CEC chez les opérés était de 93±32,2 mn et la durée moyenne du clampage aortique était de 62±25,2 mn. Les durées publiées par Al Soufi [13] et Han [10] respectivement 158.4 min et 143 min pour la CEC et 122,9 min et 115 min pour le clampage aortique sont plus longues, ceci est expliqué par une triple chirurgie valvulaire de tous leurs patients [6]. 22 patients (13,58%) de notre série ont eu un sevrage difficile de la CEC avec recours aux inotropes positifs (Dobutamine et/ou Noradrénaline) ; Des taux plus importants de recours aux inotropes positifs ont été publié dans d´autres études [6,21].

Les facteurs prédictifs d´échec de la plastie tricuspide dans notre travail sont, conformément aux autres études [15,16] l´insuffisance tricuspide préopératoire sévère, et des pressions pulmonaires postopératoires supérieures à 60mmHg. De plus, il ressort dans notre série que le rapport VD/VG télé diastolique préopératoire supérieur à 0,6 est un facteur d´échec de la plastie tricuspide. La mortalité hospitalière varie de façon importante d´une série à l´autre allant de 0,6% à 37,1% [16]. Dans notre série ce taux atteint 2,46%. La revue de la littérature montre que ce taux de mortalité est largement influencé, d´une part, par les caractéristiques démographiques des patients inclus, de l´état cardiovasculaire et, d´autre part, par la nature du geste tricuspide effectué. Ainsi, la chirurgie tricuspide est considérée comme le reflet d´une atteinte valvulaire gauche négligée. Tous les auteurs soulignent la difficulté d´apprécier la mortalité opératoire du geste tricuspide puisque la chirurgie est double: mitrale et tricuspide [16].

Certes, les résultats de notre travail sont très parlant, néanmoins quelques limites restent non négligeables: la limite principale reste le caractère rétrospectif de l´étude, ainsi que la réalisation des échocardiographies préopératoires et post opératoires par plusieurs cardiologues du service d´exploration fonctionnelle peut biaiser cette étude puisque c´est un examen opérateur-dépendant. De plus, l´analyse de la valeur de l´excursion systolique du plan de l´anneau tricuspide (TAPSE) et la valeur de l´onde S n´ont pas été possibles car les données disponibles n´étaient pas en nombre suffisant (paramètres non mentionnés dans plus de 80% des comptes rendu d´échocardiographie).

## Conclusion

Les résultats de la chirurgie tricuspide sont très variables, que ce soit en termes de succès du geste chirurgical ou de mortalité postopératoire. Ceci est essentiellement dû à la grande disparité entre les différentes séries, que ce soit dans le choix des patients, du stade évolutif de la cardiopathie au moment opératoire, du type de chirurgie tricuspide choisi et les gestes valvulaires gauches éventuels associés.

### Etat des connaissances sur le sujet

- *Les facteurs prédictifs d´échec de la plastie tricuspide dans notre travail sont, conformément aux autres études: l´insuffisance tricuspide préopératoire sévère, et des pressions pulmonaires postopératoires supérieures à 60mmHg*.

### Contribution de notre étude à la connaissance

- *Les résultats de la chirurgie tricuspide sont très variables, il ressort dans notre série que le rapport VD/VG télé diastolique préopératoire supérieur à 0,6 est un facteur d´échec de la plastie tricuspide*.
